# Image Thresholding Segmentation on Quantum State Space

**DOI:** 10.3390/e20100728

**Published:** 2018-09-23

**Authors:** Xiangluo Wang, Chunlei Yang, Guo-Sen Xie, Zhonghua Liu

**Affiliations:** 1 School of Information Technology, Luoyang Normal University, Luoyang 471934, China; 2 School of Information Engineering, Henan University of Science and Technology, Luoyang 471023, China

**Keywords:** image segmentation, thresholding, von Neumann entropy, density matrix

## Abstract

Aiming to implement image segmentation precisely and efficiently, we exploit new ways to encode images and achieve the optimal thresholding on quantum state space. Firstly, the state vector and density matrix are adopted for the representation of pixel intensities and their probability distribution, respectively. Then, the method based on global quantum entropy maximization (GQEM) is proposed, which has an equivalent object function to Otsu’s, but gives a more explicit physical interpretation of image thresholding in the language of quantum mechanics. To reduce the time consumption for searching for optimal thresholds, the method of quantum lossy-encoding-based entropy maximization (QLEEM) is presented, in which the eigenvalues of density matrices can give direct clues for thresholding, and then, the process of optimal searching can be avoided. Meanwhile, the QLEEM algorithm achieves two additional effects: (1) the upper bound of the thresholding level can be implicitly determined according to the eigenvalues; and (2) the proposed approaches ensure that the local information in images is retained as much as possible, and simultaneously, the inter-class separability is maximized in the segmented images. Both of them contribute to the structural characteristics of images, which the human visual system is highly adapted to extract. Experimental results show that the proposed methods are able to achieve a competitive quality of thresholding and the fastest computation speed compared with the state-of-the-art methods.

## 1. Introduction

Image segmentation is the task of dividing the image into different regions, each one of which ideally belongs to the same object or content. As a key step from image processing to computer vision, image segmentation is the target expression and has an important effect on the feature measurement, high-level image analysis and understanding [1,2]. Examples of image segmentation applications include medical imaging [3,4], document image analysis [5], object recognition [6,7] and quality inspection of materials [8,9]. In the last two decades, a wide variety of segmentation techniques have been developed, which conventionally fall into the following two categories [2]: layer-based and block-based segmentation methods [10,11]. Among all these techniques, the thresholding methods offer numerous advantages such as smaller storage space, fast processing and ease in manipulation.

In general, thresholding methods can be classified into parametric and nonparametric approaches [12]. Parametric approaches assume that the intensity distributions of images obey the Gaussian mixture (GM) model, which means the number and parameters of Gaussians in the mixture (the model selection) must be determined [13]. Although these problems have been traditionally solved by considering the expectation maximization (EM) algorithm [14] or gradient-based methods [15,16], the methods are time consuming. Nonparametric approaches find the thresholds that separate regions of an image in an optimal manner based on discriminating criteria such as the between-class variance [17], cluster distance [18], entropy [19,20,21,22], etc. Nonparametric methods have shown the advantage of dispensing with the modeling thresholding. However, they still suffer from the problem of high time consumption, although many techniques based on intelligent optimization algorithms (IOAs) [23,24,25] have been used to speed up the thresholding procedure.

Quantum computation and quantum information processing techniques have shown an immense potential and a revolutionary impact on the field of computer science, due to their remarkable resources: quantum parallelism, quantum interference and entanglement of quantum states. Information representing and processing in the framework of quantum theory is powerful for solving complex problems that are difficult or currently even impossible for conventional methods. The most significant works include Shor’s quantum integer factoring algorithm, which can find the secret key encryption of the RSA algorithm in polynomial time [26], and Grover’s quantum search algorithm for databases, which could achieve quadratic speedup [27]. In the recent years, quantum approaches have been introduced into the image processing field. Various quantum image representation models have been proposed, such as qubit lattice [28] and flexible representation of quantum images (FRQI) [29]. Meanwhile, several applications of quantum image processing have been researched including quantum image segmentation [30], quantum edge detection [31], quantum image recognition [32], quantum image watermarking [33] and quantum image reconstruction [34]. Though the research in quantum image processing still confronts fundamental aspects such as image representation on a quantum computer and the definition of basic processing operations, we still could be inspired to completely exploit new methods for some classical problems from a quantum information theoretical viewpoint.

In this paper, we address the thresholding problem on quantum state space. The proposed methods relate to the details of image representation by utilizing the density matrix, optimal threshold selection based on the criteria of the maximum von Neumann entropy, a novel image encoding scheme and the corresponding segmentation approaches, which can totally avoid the process of optimal solution searching. Specifically, the contributions of this paper mainly include the following aspects:

(1) We present an image thresholding method based on the criteria of global quantum entropy maximization (GQEM), which has an equivalent object function to Otsu’s, but gives more explicit physical interpretation of image thresholding in the language of quantum mechanics.
(2) The quantum lossy-encoding based entropy maximization (QLEEM) approach is proposed to deal with the time consumption problem of thresholding. The QLEEM algorithm directly takes the eigenvalues of density matrices of lossy-encoded images as segmenting clues and then avoids the time-consuming process of searching for optimal thresholds. It can achieve the highest execution speed compared with the state-of-the-art methods.
(3) Due to the physical meaning of the lossy-encoding scheme and the unique procedure of optimal thresholding, a brand-new approach to determine the upper bound of the thresholding level automatically is offered in the proposed QLEEM algorithm. For most of the existing methods, this parameter is conventionally predetermined according to empirical knowledge.
(4) The QLEEM method provides the maximum inter-class separability with lower loss of intra-class information; thus, segmented images could keep more structural information. This feature is highly consistent with the way the human visual system (HVS) works.

The paper is organized as follows: Section 2 gives a brief description of the image thresholding and introduces some state-of-the-art thresholding methods including Otsu’s between-class variance method [17], Kapur’s entropy-criterion method [19], the quantum version of Kapur’s method [35], and Tsallis entropy-based method [22]. Section 3 introduces the details of the proposed methods. Section 4 provides the experimental results and discussions about our method’s performance. The conclusions of this study are drawn in the last part of this paper.

## 2. Related Works

Thresholding is a process in which a group of thresholds is selected under some criteria, and then, pixels of an image are divided into a series of sets or classes according to the rule of:

(1) $$l\to C_{i}\;if\;th_{i-1}\leq l<th_{i},$$

where l∈[0,L−1] represents the intensity level of image pixels, $\left\{th_{i}|i=1,2,\cdots ,M-1\right\}$ is the set of thresholds and $\left\{C_{i}|i=1,2,\cdots ,M\right\}$ are classes labeling different groups of pixels.

Otsu’s between-class variance method [17] selects the optimal thresholds by maximizing the following object function:

(2) $$\sigma^{2^{c}}=\sum_{i,j}\omega_{i}\omega_{j}{\left(\mu_{i}-\mu_{j}\right)}^{2}.$$

Here, *i* and *j* index the intensity classes, and $\omega_{i}$ and $\mu_{i}$ are the probability of occurrence and the mean of a class, respectively. Such values are obtained as:

(3) $$\omega_{i}=\sum_{j=th_{i-1}+1}^{th_{i}}p_{j},\mu_{i}=\sum_{j=th_{i-1}+1}^{th_{i}}q_{j}j.$$

where $p_{j}$ denotes the probability distribution of pixels and $q_{j}=p_{j}/\omega_{i}$. As we know, Otsu’s method can achieve the best segmenting results if no contextual or semantic information is considered, but it suffers from the drawback of time-consuming searching for optimal thresholds.

Kapur presented another discriminant criterion based on maximum entropy [19]:

(4) $$\arg\max_{TH}\sum_{i=0}^{M-1}H\left(C_{i}\right).$$

where $H(C_{i})$ is the Shannon entropy corresponding to a specific class, which is defined as:

(5) $$H\left(C_{i}\right)=-\sum_{j=th_{i-1}+1}^{th_{i}}q_{j}\log q_{j}.$$

Similarly, the quantum version of Kapur’s method [35] determines the optimal thresholds by maximizing the von Neumann entropy:

(6) $$\arg\max_{TH}\sum_{i=0}^{M-1}S\left(\rho_{i}\right).$$

where:

(7) $$\rho_{i}=-\sum_{j=th_{i-1}+1}^{th_{i}}q_{j}|\theta_{j}\rangle \langle \theta_{j}|$$

is the density matrix representation of the *i*-th class and:

(8) $$S\left(\rho_{i}\right)=-tr\left(\rho_{i}\log\rho_{i}\right).$$

Recently, the Tsallis entropy-based bi-level thresholding method was proposed [22], in which the optimal threshold is given by:

(9) $$t^{*}\left(q\right)=\arg\max_{t}\left[S_{T}^{A}\left(t\right)+S_{T}^{B}\left(t\right)+\left(1-q\right)S_{T}^{A}\left(t\right)S_{T}^{B}\left(t\right)\right].$$

Here, $S_{T}^{A}\left(t\right)$ and $S_{T}^{B}\left(t\right)$ represent the Tsallis entropy for object *A* and the background *B*, respectively, and the entropic index *q* can be calculated through *q*-redundancy maximization.

The effectiveness of these entropy-based methods has been proven. However, similar to Otsu’s method, they also have the drawback of high computational complexity, which will affect the efficiency of the whole vision task.

## 3. Proposed Methods

In this section, we will start with a new method, which utilizes the criteria of global quantum entropy maximization to achieve optimal thresholding, and then propose a novel encoding scheme. Based on this scheme, the improved method for thresholding is derived, which can determine optimal thresholds with linear time complexity.

### 3.1. Thresholding Based on Global Quantum Entropy Maximization

For an image, we can represent its histogram with the following entangled state of a composite quantum system:

(10) $$|I\rangle =\sum_{i=0}^{L-1}\sqrt{p_{i}}|\theta_{i}\rangle ⊗|i\rangle .$$

where we encode the *i*-th intensity level to the vector $|\theta_{i}\rangle =cos\theta_{i}|0\rangle +sin\theta_{i}|1\rangle$, which belongs to the state space of the first one-qubit subsystem (labeled as “*A*”), by establishing a bijective relationship between them, namely:

(11) $$\theta_{i}=\frac{\pi }{2}\cdot \frac{i}{L-1},i\in \left[0,L-1\right],$$

and $|i\rangle$ is the computational basis state of the second subsystem (labeled as “*B*”), which denotes the indices of pixel intensities. Though $|I\rangle$ is a pure state, the subsystem *A* or *B* is in a mixed state. Therefore, we describe these quantum systems in the language of the density matrix. Assuming $|I\rangle$ is rewritten as $\rho^{AB}$, then the reduced density matrix for the subsystem *A* can be defined by:

(12) $$\begin{array}{cc} \rho & =tr_{B}\left(\rho^{AB}\right)\\ & =\sum_{i=0}^{L-1}p_{i}|\theta_{i}\rangle \langle \theta_{i}|. \end{array}$$

The density matrix ρ contains the information about the distance between any two intensities, as well as their probability distribution. This property will be very useful for thresholding.

If pixels of an image are divided into *M* classes by using *M*-1 thresholds, we represent the histogram of the segmented image with:

(13) $$|I^{\prime }\rangle =\sum_{i=0}^{M-1}\left({\sqrt{\omega }}_{i}|{\tilde{\theta }}_{i}\rangle ⊗\sum_{j=th_{i-1}+1}^{th_{i}}\sqrt{q_{j}}|i\rangle \right),$$

where ${\tilde{\theta }}_{i}=\frac{\pi }{2}\cdot \frac{\mu_{i}}{L-1}$, $\omega_{i}$ and $\mu_{i}$ are defined in Equation (3). Then, the density matrix of the subsystem *A* becomes:

(14) $$\rho^{\prime }=\sum_{i=0}^{M-1}\omega_{i}|{\tilde{\theta }}_{i}\rangle \langle {\tilde{\theta }}_{i}|,$$

and the von Neumann entropy of $\rho^{\prime }$:

(15) $$\begin{array}{cc} S(\rho^{\prime }) & =-tr(\rho^{\prime }\log\rho^{\prime })\\ & =-\lambda_{1}\log\lambda_{1}-\lambda_{2}\log\lambda_{2} \end{array}$$

can quantify how much information is retained in the segmented image; where $\lambda_{1}$ and $\lambda_{2}$ are the eigenvalues of $\rho^{\prime }$. As a result, we maximize it to determine the optimal thresholds:

(16) $$TH_{op}=\arg\max_{TH}S\left(\rho^{\prime }\right).$$

According to Equations (14) and (15), the following equation is established through simple algebraic computations:

(17) $$\lambda_{1}\lambda_{2}=\frac{1}{2}\sum_{i=0,j=0}^{M-1}\omega_{i}\omega_{j}sin^{2}\left({\tilde{\theta }}_{i}-{\tilde{\theta }}_{j}\right),$$

where $\lambda_{1}+\lambda_{2}=1$, as the restriction must be held.

It is worthwhile to note that Equation (17) can also be used to evaluate thresholding: when Equation (17) takes the maximum value, $\lambda_{1}$ and $\lambda_{2}$ will be most similar to each other, and then, $S(\rho^{\prime })$ also reaches its best value. Meanwhile, Equation (17) indicates that the distance between intensities $sin^{2}\left({\tilde{\theta }}_{i}-{\tilde{\theta }}_{j}\right)$, as well as the probability distribution ($\omega_{i}$, $\omega_{j}$) affect the thresholding results.

Different from Kapur’s entropy-based method and its quantum version, our method has more explicit physical meaning for thresholding in terms of the following features:

(1) Encoding pixel intensities on the state space of a one-qubit system can be considered as a process in which independent intensities are squeezed into a two-dimensional space. The similarity between different state vectors, as well as its probability distribution, can be described with the density matrix. Both factors contribute to thresholding.
(2) According to the fundamental principles of information theory, the image segmenting process will causes the decrease of the information contained in images. Shannon entropy cannot directly be used to measure the information losses because it quantifies the amount of information on spaces with different dimensionality for original and segmented images. On the contrary, our method encodes the histograms of original and segmented images on the same quantum state space, which indicates that their entropies are comparable. As a result, the trivial solutions for segmentation, for example the thresholds equally dividing intensities into clusters with the same probability, could never appear since the entropy of the original image acts as the upper bound of our object function for all possible solutions.
(3) From Equation (17), we find that the object function of our method is very similar to Otsu’s, described in Equation (2). The following experimental results will prove that they both achieve the best thresholding.

### 3.2. Quantum Lossy-Encoding-Based Entropy Maximization Method

As we have seen in Section 3.1, the proposed thresholding method derived from the viewpoint of quantum principles can achieve the best segmenting results similar to Otsu’s. However, it still suffers from the efficiency problem of searching for optimal thresholds. In this subsection, we present another way for image thresholding on the quantum state space.

#### 3.2.1. Quantum Lossy Encoding of Images

Different from the precedent method, we map the pixel intensities to quantum state vectors according to the following rules:

(1) Multiple qubits should be required for encoding intensity levels in accordance with the prospective number of thresholds. In other words, the state vectors are supposed to belong to an *M*-dimensional space if we want the *M*-level segmentation.
(2) The angle parameter of state vectors ranges from zero to M·π instead of π/2. Namely, $\theta_{i}=M\pi i/L$.
(3) After encoding, the terms contributing to density matrices should follow a π-periodic cyclical pattern. Namely, $|\theta \rangle \langle \theta |=|\theta +\pi \rangle \langle \theta +\pi |$.

Rule (1) provides the foundation for dividing pixel intensities into *M* classes, being linearly independent of each other. Rules (2) and (3) indicate that all state vectors representing pixel intensities are equally divided into *M* classes, and the corresponding density matrix:

(18) $$\tilde{\rho }=\sum_{i=0}^{N-1}\left(\sum_{j=0}^{M-1}p_{N\cdot j+i}\right)|\theta_{i}\rangle \langle \theta_{i}|,\theta_{i}\in \left[0,\pi \right],N=L/M,$$

only measures the information related to the local or intra-class uncertainty contributed by those adjoining intensity levels, but removes the global or inter-class information provided by those intensities far apart from each other.

**Figure 1:**
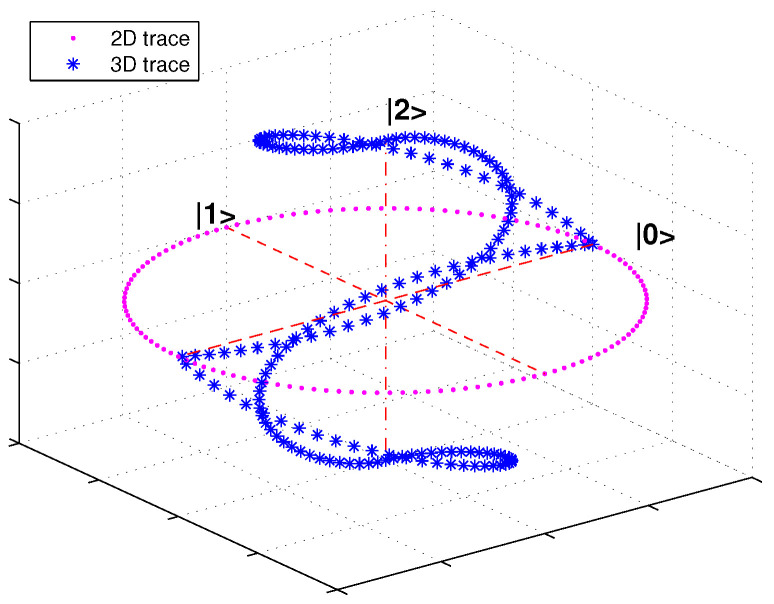
Traces of encoded state vectors on 2D and 3D space.

According to the above rules, an alternative encoding scheme is given in the recursive form of:

(19) $$\begin{array}{c} {|\theta_{i}\rangle }^{2}=cos\theta_{i}|0\rangle +sin\theta_{i}|1\rangle ,\theta_{i}=2\pi i/L\\ {|\theta_{i}\rangle }^{3}=cos\theta_{i}cos2\theta_{i}|0\rangle +cos\theta_{i}sin2\theta_{i}|1\rangle +sin\theta_{i}|2\rangle ,\theta_{i}=3\pi i/L\\ \cdots \\ {|\theta_{i}\rangle }^{M}=cos\theta_{i}{|2\theta_{i}\rangle }^{M-1}+sin\theta_{i}|M-1\rangle ,\theta_{i}=M\pi i/L \end{array}$$

where the superscript *M* is temporarily borrowed to label the dimensionality of state vectors and i∈[0,L−1] denote pixel intensities. As an example, the traces of encoded state vectors in the 2D and 3D case are shown in Figure 1.

Differing from ordinary encoding practices, the proposed scheme records local information of images, but removes the global information. More precisely, the following evidence could be verified in the 2D case: we divide intensity levels into two classes equally and equivalently quantify the amount of information with the product of eigenvalues of $\tilde{\rho }$:

(20) $$\begin{array}{cc} \lambda_{1}\lambda_{2} & =\frac{1}{2}\sum_{i=0,j=0}^{L-1}p_{i}p_{j}sin^{2}\left(\theta_{i}-\theta_{j}\right)\\ & =\frac{1}{2}\left(\sum_{i=0,j=0}^{L/2-1}p_{i}p_{j}sin^{2}\left(\theta_{i}-\theta_{j}\right)+\sum_{i=L/2,j=L/2}^{L-1}p_{i}p_{j}sin^{2}\left(\theta_{i}-\theta_{j}\right)\right)+\sum_{i=0}^{L/2-1}\sum_{j=L/2}^{L-1}p_{i}p_{j}sin^{2}\left(\theta_{i}-\theta_{j}\right). \end{array}$$

We note that the first term on the right of Equation (20) measures the local information (intra-class uncertainty) contributed by intensities in the same class, and the second term counts the global information (inter-class uncertainty) provided by intensities in different classes. Meanwhile, it is easy to verify that the values of the two terms will increase and decrease respectively when θ covers [0,2π] instead of [0,π/2].

#### 3.2.2. The QLEEM Method

Intuitively, the intensities far apart from each other and their probability distribution provide the evidence of thresholding. Therefore, we rewrite the density matrix of the given histogram in a decomposed form:

(21) $$\rho =\upsilon_{1}\rho_{1}+\upsilon_{2}\rho_{2}.$$

where $\rho_{1}$ and $\rho_{2}$ describe the probability distributions of local and remote intensity levels (that is, intra-class and inter-class uncertainty), respectively. Meanwhile, as there is no more knowledge about $\upsilon_{1}$ and $\upsilon_{2}$ except $\upsilon_{1}+\upsilon_{2}=1$, we assume $\upsilon_{1}=\upsilon_{2}=1/2$ according to the foundational principle of the entropy theory.

Now, we substitute $\rho_{1}$ with $\tilde{\rho }$ given by the proposed lossy encoding scheme, since it contains the information contributed by local uncertainty of intensity vectors, and maximize the von Neumann entropy of Equation (21) for determining optimal thresholds:

(22) $$TH_{op}=\arg\max S\left(\frac{1}{2}\tilde{\rho }+\frac{1}{2}\hat{\rho }\right).$$

Here, we adopt orthogonal state vectors in *M*-dimensional space representing *M* classes after thresholding, since we want these intensity classes to be as independent as possible. Let:

(23) $$\tilde{\rho }=\sum_{i=0}^{M-1}\lambda_{1,i}|\theta_{1,i}\rangle \langle \theta_{1,i}|,\hat{\rho }=\sum_{i=0}^{M-1}\lambda_{2,i}|\theta_{2,i}\rangle \langle \theta_{2,i}|$$

be orthonormal decompositions for the states $\tilde{\rho }$ and $\hat{\rho }$, then for any one eigenvector of $\tilde{\rho }$ denoted with $|\theta_{1,j}>$, there must exist an eigenvector of $\hat{\rho }$ named $|\theta_{2,i}>$ satisfying the relationship of $|\theta_{2,i}>=\pm |\theta_{1,j}>$ when $S(\left(\tilde{\rho }+\hat{\rho }\right)/2)$ takes the max value. Meanwhile, the eigenvalues of the state can be determined according to the following equation:

(24) $$\lambda_{2,i}=\frac{2}{M}-\lambda_{1,j},if|\theta_{2,i}\rangle =\pm |\theta_{1,j}\rangle .$$

For the sake of representation, here we give the evidence of the above conclusion for the 2D situation. Assuming $\lambda_{1}$ and $\lambda_{2}$ are eigenvalues of the state $(\tilde{\rho }+\hat{\rho })/2$, its entropy will take the maximum value if we equivalently maximize:

(25) $$\begin{array}{cc} \lambda_{1}\lambda_{2}= & \lambda_{1,0}\lambda_{2,0}sin^{2}\left(\theta_{1,0}-\theta_{2,0}\right)+\lambda_{1,0}\lambda_{2,1}sin^{2}\left(\theta_{1,0}-\theta_{2,1}\right)+\lambda_{1,1}\lambda_{2,0}sin^{2}\left(\theta_{1,1}-\theta_{2,0}\right)\\ & +\lambda_{1,1}\lambda_{2,1}sin^{2}\left(\theta_{1,1}-\theta_{2,1}\right)+\lambda_{1,0}\lambda_{1,1}+\lambda_{2,0}\lambda_{2,1} \end{array}$$

Notice that $\langle \theta_{1,0}|\theta_{1,1}\rangle =0$ and $\langle \theta_{1,0}|\theta_{1,1}\rangle =0$ must hold. Then:

(26) $$\begin{array}{cc} \lambda_{1}\lambda_{2}= & \left(\lambda_{1,0}\lambda_{2,0}+\lambda_{1,1}\lambda_{2,1}\right)sin^{2}\left(\theta_{1,0}-\theta_{2,0}\right)+\left(\lambda_{1,0}\lambda_{2,1}+\lambda_{1,1}\lambda_{2,0}\right)cos^{2}\left(\theta_{1,0}-\theta_{2,0}\right)\\ & +\lambda_{1,0}\lambda_{1,1}+\lambda_{2,0}\lambda_{2,1} \end{array}$$

will take the extremum when $\langle \theta_{1,0}|\theta_{1,1}\rangle =0\;or\;1$. In other words,

(27) $$\left\{\begin{array}{c} |\theta_{2,0}\rangle =\pm |\theta_{1,0}\rangle \\ |\theta_{2,1}\rangle =\pm |\theta_{1,1}\rangle \end{array}\right.or:\;\left\{\begin{array}{c} |\theta_{2,0}\rangle =\pm |\theta_{1,1}\rangle \\ |\theta_{2,1}\rangle =\pm |\theta_{1,0}\rangle \end{array}\right.$$

must hold. Without loss of generality, we adopt the first case of Equation (27) for the succeeding discussions. Then:

(28) $$\begin{array}{cc} \lambda_{1}\lambda_{2} & =\lambda_{1,0}\lambda_{2,1}+\lambda_{1,1}\lambda_{2,0})+\lambda_{1,0}\lambda_{1,1}+\lambda_{2,0}\lambda_{2,1}\\ & =-\lambda_{2,0}^{2}+\left(1+\lambda_{1,0}-\lambda_{1,1}\right)\lambda_{2,0}+\lambda_{1,0}\lambda_{1,1}+\lambda_{1,1} \end{array}$$

will reach its maximum value when:

(29) $$\begin{array}{cc} \lambda_{2,0} & =(1+\lambda_{1,1}-\lambda_{1,0})/2\\ & =\frac{2}{M}-\lambda_{1,2}\\ & =\lambda_{1,1} \end{array}.$$

The above conclusions have the instructive function for thresholding, which can be seen in two aspects:

(1) Based on the proposed lossy-encoding scheme, we can directly calculate the eigenvalues of $\hat{\rho }$ according to Equation (24), which represent the probability distribution of intensity classes after thresholding, and then determine the optimal threshold values.
(2) As the probability with which any one intensity class occurs must be greater than zero, according to Equation (24), all eigenvalues of the density matrix $\tilde{\rho }$ would satisfy the condition of $\lambda_{1,i}<2/M$. Otherwise,$\lambda_{1,i}\geq 2/M$ indicates that there exist meaningless and unnecessary classes for segmentation. In summary, the upper bound of the thresholding level can be determined using our method. This feature implies that our method is more feasible than the most of the other existing ones, since the thresholding level, as a hyperparameter, is often predetermined empirically.

Finally, the optimal thresholds $TH=th_{1},th_{2},\cdots ,th_{M-1}$ can be determined according to the following relationships:

(30) $$\left\{\begin{array}{c} \sum_{i=0}^{th_{1}}p_{i}\leq \lambda_{0}<\sum_{i=0}^{th_{1}+1}p_{i}\\ \cdots \\ \sum_{i=th_{M-2}+1}^{th_{M-1}}p_{i}\leq \lambda_{M-1}<\sum_{i=th_{M-2}+1}^{th_{M-1}+1}p_{i} \end{array}\right.$$

where $\lambda_{0},\lambda_{1},\cdots ,\lambda_{M-1}$ is the sequence taken from the eigenvalue set of $\hat{\rho }$, and the corresponding sequence $|\theta_{0}\rangle ,|\theta_{1}\rangle ,\cdots ,|\theta_{M-1}\rangle$ belongs to the circular permutation of all eigenvectors, which satisfy the following rules:

(31) $$\left\{\begin{array}{c} |\theta_{i}\rangle =\arg\max_{j}\left|\langle \theta_{j}|0\rangle \right|\\ |\theta_{(i+1)modM}\rangle =\arg\max_{j}\left|\langle \theta_{j}|1\rangle \right|\\ \cdots \\ |\theta_{(i+M-1)modM}\rangle =\arg\max_{j}\left|\langle \theta_{j}|M-1\rangle \right| \end{array}\right..$$

According to the methods mentioned above, the framework of the QLEEM algorithm is given in Algorithm 1.

**Algorithm 1** The framework of the QLEEM algorithmInput: The original image *I*, the thresholding level *M* Output: The optimal thresholds Init: Compute the histogram of the input image; Step 1: Obtain density matrix $\tilde{\rho }$ by using the lossy-encoding scheme; Step 2: Calculate the eigenvalues and eigenvectors of $\tilde{\rho }$ and then $\hat{\rho }$ Step 3: Enumerate all possible *M* circular sequences of the eigenvalues of $\hat{\rho }$, and then get *M* groups of thresholds; Step 4: loop over the *M* groups of thresholds, and select the optimal one based on which the entropy denoted in Equation (15) takes the maximum value.

#### 3.2.3. Time Complexity of the QLEEM Algorithm

For the problem of *M*-level thresholding segmentation of images containing *L*-level intensities, the time of calculating the density matrix $\tilde{\rho }$ is O(L); computing eigenvalues and eigenvectors of $\tilde{\rho }$ needs $O(M^{3})$; the time for performing Step 3 is O(M!+L); and the loop in Step 4 consumes $O(M*2^{3})$ time. Since M<<L is satisfied in general cases, the optimal performance time of the QLEEM algorithm is achieved by T=O(L), which notably outperforms Otsu’s $T=O(A_{L-1}^{M-1}/2^{M-2})$.

## 4. Experiments and Comparisons

### 4.1. Datasets and Settings

To evaluate the performance of the proposed methods, a set of standard test images was obtained from the Berkeley segmentation dataset [36]. All of the test images are 8-bit in depth, with a size of 481 × 321 pixels. The algorithms used for comparison are Otsu’s between-class variance method [17], Kapur’s entropy criterion method [19], the quantum version of Kapur’s [35] and our GQEM and QLEEM methods. These algorithms are implemented with MathWorks MATLAB 2014a on a Thinkpad notebook with an Intel Core-i5 2.2-GHz processor, 16 GB RAM and Ubuntu 14.04.

Threshold levels, quality of segmented images and time complexity are the most important indicators for evaluating the performance of image thresholding algorithms. Here, we evaluate the quality of segmented images by using the peak signal-to-noise ratio (PSNR) and structural similarity (SSIM). In addition, four measures: the Dice similarity coefficient (DICE) [37], the probabilistic rand index (PRI) [38], the global consistency error (GCE) [36] and the variation of information (VI) [39], are used to assess segmentations against ground truth data. Time complexity is measured by the execution time required in these methods. In particular, except for the proposed QLEEM, all the other exhaustive-search-based methods used in our experiments are sped up with the harmony search multithresholding algorithm (HSMA) [25].

### 4.2. Experimental Results and Comparisons

**Figure 2:**
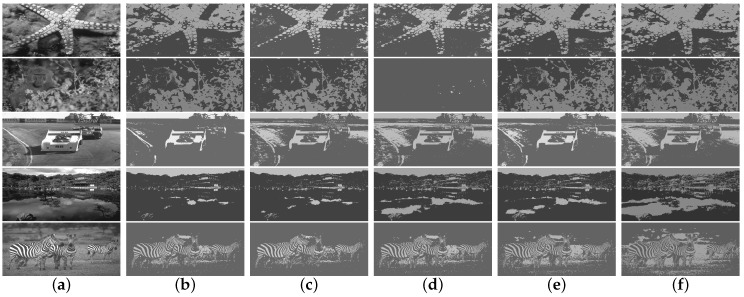
Visual comparison of (**a**) original images and bi-level segmented ones by using the (**b**) Otsu, (**c**) Kapur, (**d**) quantum version of Kapur’s method (QKapur), (**e**) global quantum entropy maximization (GQEM) and (**f**) quantum lossy-encoding-based entropy maximization (QLEEM) methods, respectively.

**Figure 3:**
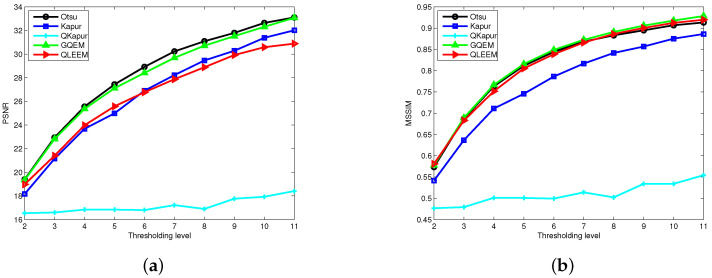
Quality assessment of the segmented images in terms of (**a**) peak signal-to-noise ratio (PSNR) and (**b**) structural similarity (SSIM).

We applied these algorithms to all 300 pictures contained in the standard test dataset for assessing their performance. For the sake of representation, only five images, which are presented in Figure 2, have been used to show the bi-level segmented results. In Figure 3, the thresholding quality of the outcomes is analyzed considering the complete set, where the PSNR and SSIM scores are calculated under different thresholding levels, and we take the average values on the whole dataset.

**Figure 4:**
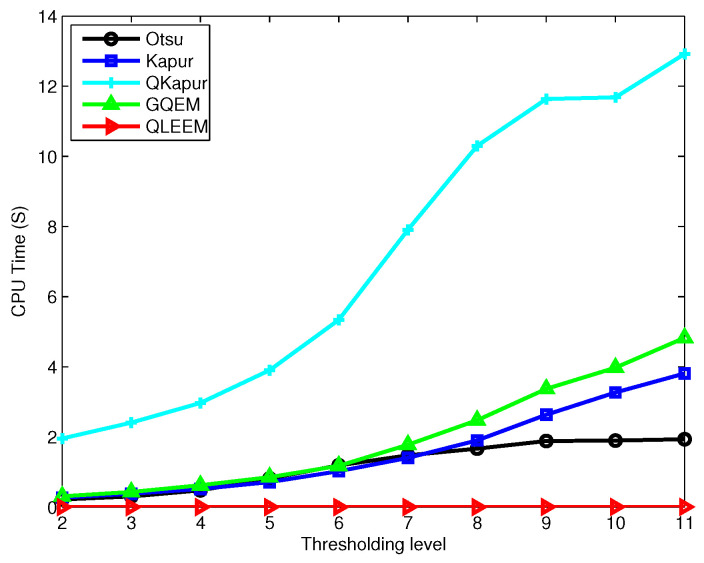
The comparison of the time consumption of different methods under different thresholding levels.

Meanwhile, we recorded the CPU time consumed by these algorithms, and the average values for all the test images under different thresholding levels are depicted in Figure 4. As an example, the experimental results in terms of thresholding level, thresholds and CPU time are tabulated in Table 1 for a randomly selected image.

From Figure 2, we find that the segmentations obtained by using GQEM, QLEEM and Otsu are visually indistinguishable, which means these three methods have a similar performance. This conclusion can be further confirmed in Figure 3: the GQEM method obtains almost the same PSNR score as Otsu’s in spite of very little computational error; meanwhile, both GQEM and QLEEM outperform the others in terms of SSIM. The experimental results can be explained with the criteria of maximizing quantum entropy and the lossy-encoding scheme proposed in our methods, because they emphasize the weight of between-class variance and retain the local information, respectively. This feature is highly consistent with the SSIM method, which assesses the perceived quality of images based on structural similarity indicators, such as contrast and local inter-dependencies of pixels.

Examining Figure 4 and Table 1, we can see that the proposed QLEEM algorithm achieves the fastest execution speed (at least 100-times faster than Otsu in the case of bi-level thresholding and up to 350-times when the number of thresholds increases to five). In addition, the time consumption of QLEEM was insensitive to increments of the threshold level, since the complexity of our algorithm was mainly correlated with the total intensity level, instead of the amount of thresholds.

**Figure 5:**
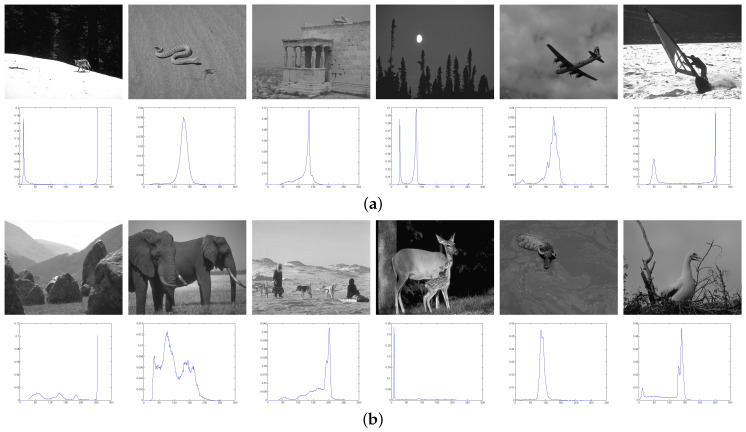
Two groups of images in the test dataset, to which the QLEEM algorithm suggests applying (**a**) bi-level and (**b**) tri-level thresholding, respectively.

On the other hand, the upper bounds of the thresholding level recommended by the proposed QLEEM algorithm were tested. We found that the maximum possible amount of thresholds was lower than 10 for about 40 images in the test set. Our algorithm would terminate when we try to apply more thresholds to them. Figure 5 lists two groups of images and corresponding histograms, for which the proposed algorithm gave one and two thresholds, respectively. According to the visual observation, it is reasonable to believe that the suggested amounts of thresholds are feasible, as there are no more than three distinct peaks in their histograms.

**Figure 6:**
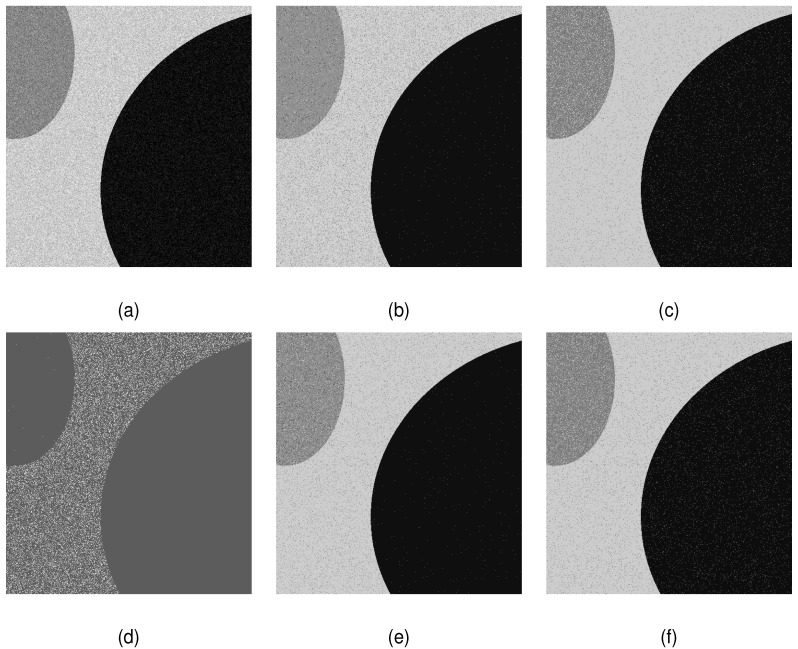
Comparison of segmentation results on a synthetic image. (**a**) noisy image (Gaussian noise with zero mean and 3% variance); (**b**) Otsu result; (**c**) Kapur result; (**d**) QKapur result; (**e**) GQEM result; (**f**) QLEEM result.

Finally, we evaluate segmentations against the ground truth data. The first experiment is performed on a synthetic image corrupted by Gaussian noise (the mean value is zero, and the variance is 0.03), which is utilized for testing the efficiency and robustness of the proposed methods. Figure 6 shows the noisy image and segmentation results obtained by different algorithms. In addition, the performance indexes: the DICE ratio, PRI, GCE and VI scores, are used to assess the robustness of these algorithms. The corresponding scores are listed in Table 2.

The visual comparison in Figure 6 shows that the proposed GQEM and QLEEM algorithms produce clearer and more accurate segmentation results. From Table 2, we can confirm this conclusion: our GQEM clearly outperformed the others on the DICE, PRI, GCE and VI values. The robustness of the proposed GQEM for noisy images can be explained by comparing the object function of GQEM and Otsu. Considering the last term in Equations (2) and (17), both of them measure the distance between pixel intensities, but our GQEM method scaled the range [0,L−1] of this parameter down to [0,1]. This feature is helpful for suppressing the high contrast caused by noise, and then, our GQEM algorithm partly played the role of a low-pass filter in segmentation tasks.

In the second experiment, we performed thresholding segmentation on BSDS300 dataset and compared the results with the ground truth segmentations in terms of the DICE, PRI, GCE and VI indexes. The average scores of these indicators obtained by different algorithms are presented in Table 3.

From Table 3, we can see that all the listed algorithms obtained lower scores compared with those that have been well trained with the manually-labeled dataset. In general, thresholding segmentation is a form of unsupervised segmentation, which cannot use any a priori knowledge involving the ground truth of a training set of images. Furthermore, the proposed GQEM and QLEEM along with the others used for comparison are all histogram-based algorithms. They achieve optimal segmentation by merely utilizing the probability distribution of colors, instead of the spatial and texture information.

## 5. Conclusions

In this paper, we address the image thresholding problem on quantum state space. The proposed GQEM and QLEEM methods follow a different way to represent images and determine the optimal thresholds in the language of quantum mechanics. In summary, the contributions of this paper mainly include the following aspects: (1) To our knowledge, this is the first application of the global quantum entropy criteria to the thresholding problem. The von Neumann entropy is more powerful for image segmentations than the Shannon entropy, because it measures the distance between pixel intensities, as well as the probability distribution. (2) Compared with other state-of-the-art approaches, our QLEEM algorithm tends to retain more structural information after segmentations. It is highly consistent with the way in which the HVS works. (3) The proposed QLEEM algorithm has the lowest consumption of execution times known to us, even compared with others that are sped up with some intelligent optimization techniques.

## Figures and Tables

**Table 1:** Performance comparison in terms of thresholding level (*M*), thresholds and computation time.

Method	*M*	Thresholds	CPU Time (s)
Otsu	2	116	0.233523
	3	85-157	0.313405
	4	69-120-178	0.348805
	5	60-101-138-187	0.666153
	6	52-85-117-150-193	1.293793
Kapur	2	155	0.256437
	3	91-170	0.395228
	4	75-130-183	0.473902
	5	66-113-160-203	1.222006
	6	56-93-132-170-209	1.181965
QKapur	2	147	2.007029
	3	10-147	2.12888
	4	10-17-147	3.924715
	5	10-17-147-252	3.114482
	6	10-17-147-251-252	4.59602
GQEM	2	114	0.338808
	3	84-147	0.410247
	4	70-117-168	0.666514
	5	62-99-133-176	0.682051
	6	54-86-114-143-182	0.985941
QLEEM	2	107	0.001661
	3	86-135	0.002079
	4	62-106-153	0.002549
	5	53-90-121-160	0.003043
	6	49-83-106-133-166	0.003673

**Table 2:** Performance of different algorithms on a noisy image (the best values are highlighted). DICE, Dice similarity coefficient; PRI, probabilistic Rand index; GCE, global consistency error; VI, variation of information.

Algorithm	DICE	PRI	GCE	VI
Otsu	0.889787	0.934784	0.09807	0.54778
Kapur	0.908592	0.946141	0.093275	0.532568
QKapur	0.472366	0.426367	0.084447	1.570079
GQEM	***0.921509***	***0.955491***	***0.078646***	***0.45552***
QLEEM	0.908281	0.948501	0.097511	0.580201

**Table 3:** Average performance of different algorithms on BSDS300 dataset (the best values are highlighted).

Algorithm	DICE	PRI	GCE	VI
Otsu	0.411934	0.613044	0.385938	2.825647
Kapur	0.400079	***0.64313***	0.366348	2.49384
QKapur	0.363979	0.542463	***0.1704***	***1.802242***
GQEM	***0.412396***	0.611379	0.384827	2.892085
QLEEM	0.405824	0.614035	0.386781	2.931183
